# The comparison of the applicability of low-frequency laser, β-tricalcium phosphate, platelet-rich fibrin, and bone marrow in the treatment of tibial fractures in rabbits

**DOI:** 10.1590/acb407525

**Published:** 2025-10-17

**Authors:** Umut Alpman, Gultekin Atalan, Efe Karaca, Gokcen Perk

**Affiliations:** 1 University of Erciyes – Faculty of Veterinary Medicine – Department of Surgery – Kayseri – Turkey.; 2 Ondokuz Mayıs University – Faculty of Veterinary Medicine – Department of Veterinary Pathology – Samsun – Turkey.

**Keywords:** Bone and Bones, Transplants, Lasers, Regeneration

## Abstract

**Purpose:**

To compare and evaluate the radiologic, clinical, and histopathologic results of the treatment methods applied in diaphyseal tibial fractures.

**Methods:**

A complete tibial fracture was created in the tibial diaphysis in each rabbit. Experimentally generated fracture fragments were fixed by intramedullary pinning. In the control group (group I), the bone fracture area was left to heal without any treatment technique. Group II received low-energy laser therapy once daily to the surgical side for 30 days; group III, autologous bone marrow aspirated from the left proximal tibia; group IV, a combination of bone marrow obtained by aspiration and synthetic β-tricalcium phosphate (β-TCP); and in group V, a platelet-rich fibrin (PRF) membrane obtained from the central auricular artery was applied to the fracture side.

**Results:**

In the X-ray analysis, it was determined that group IV had the fastest score increase, while group I had the lowest scores. In group IV, no lameness that persisted until the end of the study was observed in any rabbit. When the histopathological scores of the different groups were examined, it was seen that the lowest score belonged to group I, and the highest score was in group IV.

**Conclusion:**

The highest rate of new bone formation and bone regeneration was achieved when the combination of aspirated bone marrow and β-TCP granules was applied. The experimental group with PRF membrane application exhibited the least osteogenic characteristics among all experimental groups.

## Introduction

Platelet-rich fibrin (PRF) is a widely used biomaterial in bone regeneration as a second-generation platelet concentrate containing growth factors and cellular components (platelets, leukocytes, cytokines) that promote osteogenic cell migration^1^^–^ 3. Many studies focused on PRF due to its potential to accelerate the healing process in fractures^4^.

Low-energy laser therapy is widely used in bone engineering to promote bone healing with its non-invasive and biocompatible nature^5^^,^ 6. However, the impact of low-energy laser application on fractures are still debatable due to previously published distinct and contradicting results^7^.

β-tricalcium phosphate (β-TCP) is a synthetic graft material that promotes bone healing with its high osteoconductivity and biocompatibility^8^^,^ 9. However, its osteoinductive capacity has been reported to be lower than autologous or allogeneic grafts^10^.

Bone marrow is rich in cytokines and mesenchymal stem cells and is used as an autograft or by injection to promote bone regeneration^11^. However, it is not sufficient by itself due to its lack of osteoconductive scaffolding. Therefore, its use in combination with bone substitutes such as β-TCP is recommended^12^.

Comparative analysis of the effects of treatment modalities such as biomaterials, grafts, and laser applications on fracture healing contributes to the development of more effective and reliable clinical treatment strategies^13^^,^ 14. The four different treatment modalities compared in this study affect different biological processes of fracture healing. β-TCP provides osteoconductive support by providing a physical scaffold^8^^,^ 9, while PRF provides biochemical stimulation through growth factors^1^^–^ 3. Bone marrow provides regeneration at the cellular level with direct osteogenic cell contribution^11^, and the low-energy laser noninvasively supports the healing process through biostimulation by triggering cellular functions^5^^,^ 6.

The used treatment approaches target a different aspect of fracture healing and have different mechanisms of action and application methods. The treatment modalities used in this study were included in the study considering their preference in clinical practice, conflicting findings in the literature in terms of outcomes, potential therapeutic efficacy, and experimental applicability.

In our study, we aimed to investigate the effects of treatment approaches affecting different biological processes on bone regeneration, fracture healing, and new bone formation, and to contribute to clinical practice.

## Methods

This study was conducted with the approval of Erciyes University Animal Experiments Local Ethics Committee (decision no. 19/075, of March 16, 2019).

### Animal material

A total of 40, 6-month-old, male New Zealand White rabbits weighing 2–4 kg (2.243 ± 0.67) each were used for the presented study. Rabbits were housed in single cages with standard cage dimensions of 60–75-cm width, 75-cm length, and 45–60-cm height at Erciyes University Experimental Research, Application and Research Center. Rabbits were quarantined for one week before the study. During this period, feed consumption, urination, feces, heart, respiration, pulse rate, and body temperature were checked daily, and healthy rabbits were used as study material. During the study, all rabbits were kept in single cages. Rabbits were fed *ad-libitum* and were capted under regular conditions and exposed to 12 hours of light and 12 hours of darkness, at 21 ± 1°C ambient temperature and 50 ± 1% humidity throughout the study.

### Anesthesia protocol

As a pre-anesthetic, 5 mg/kg xylazine-HCL (Rompun 2%, Bayer, Kiel, Germany) was administered intramuscularly (IM) to each rabbit, and 35 mg/kg kematine-HCL (Ketasol 10%, Richter Pharma AG, Wels, Austria) was administered IM for the anesthesia induction after pre-anesthetic. The general anesthesia was maintained during the operation with 2–3% sevoflurane (Sevorane Liquid 100%, AbbVie, Queenborough, England) by connecting the rabbits to inhalation anesthesia equipment (ANS 200 V Graphic Screen Anaesthesia Workstation, Atese Medikal, Ankara, Turkey). The rabbits were monitored regularly throughout the operation to prevent complications during anesthesia.

### Tibial fracture formation

A 3-cm long mediolateral (ML) skin incision was carried out on the right leg of the tibia region. In each rabbit, a standardized surgical procedure was performed to create an equal fracture in the tibia. The cranial tibial muscle and the medial part of the flexor digitorum profundus muscles were released by incising the crural fascia under the skin. The corpus tibiae were exposed by retracting the muscles. The saphenous artery and medial saphenous vein network were protected carrefully. A fracture was created in the cortical bone of the corpus tibia using a 5-mm bone saw^6^^,^ 15. The fractured side was stabilized with the application of a 3-mm titanium intramedullary pin. Following that, graft material was applied to research groups.

The control group (group I) did not receive any further treatment other than an intramedullary pin throughout the entire study. After each procedure, all layers were closed by a simple continuous suture pattern with a 2/0 multifilament polyglycolic acid suture.

### Laser application: group II (n = 8)

Starting with the first day of the procedure, low-energy laser application was performed once daily on the surgical side for 30 days. The settings were adjusted to 10-J/cm2 energy density, 200-mW power, 830-nm wavelength, and 3-Hz frequency. Treatment was applied for 5 minutes to the 3.5-cm^2^ cross-sectional area of the tibial fracture region by using a low-energy laser equipment (Maestro CMM, Maestro Laser Professional Laser Therapy Equipment, MediCom, Czech Republic)^16^.

### Bone marrow aspiration and application: group III (n = 8)

The bone marrow aspiration was performed from the medial proximal tibia. The knee (*articulatio genu*) joint was positioned at the 90-degree flexion position. An 18-gauge bone marrow aspiration needle was inserted into this location at a 45-degree angle towards the tibial medulla. After reaching the medulla, the trocar tip of the needle was removed, and a sterile injector was placed on the aspiration needle. One mL of bone marrow was aspirated into the injector. Collected bone marrow cells were kept in the syringe for 15 minutes to obtain a concentrate. Following the preparation of the concentrate, the whole amount of the bone marrow aspirate concentrate was injected into the fracture site^17^^–^ 19.

### Application of β-tricalcium phosphate: group IV (n = 8)

The bone marrow aspiration was applied to the rabbits of group IV with the β-TCP (Suprabone, BMT Calsis, Ankara, Turkey) application. After the aspiration, about 2 g of granular, radiopaque, and resorbable β-TCP bone graft material was placed in a sterile container. One mL of aspirated bone marrow was added to the β-TCP bone graft in the sterile container and dissolved with sterile forceps. The mixture was stirred continuously until a homogeneous composition was achieved. The obtained bone marrow and β-TCP graft material mixture was transplanted to the experimentally induced tibial fracture side^20^.

### Application of platelet-rich fibrin material: group V (n = 8)

For PRF membrane application, 5 cc of arterial blood were drawn from the central auricular artery. The blood was collected into the tubes without an anticoagulant for PRF production. Attained blood samples were centrifuged for 10 minutes at 3,000 rpm. The PRF membrane layer located in the middle of the tube was taken from the tube using flat forceps. Following that, it was placed between gauze pads and rinsed with sterile saline to attain the dense section. The PRF membrane was directly applied to the surroundings of the fracture site^21^.

### Postoperative procedures

A support dressing was applied to the right hind leg of rabbits in all groups after the operation. A windowed support dressing application with access to the incision line was preferred. During the first week after the operation, the surgical wounds of the rabbits were monitored for signs of infection. Daily wound dressing using polyvinyl-pyrrolidone-iodine (Batticon Adeka, Samsun, Turkey) and oxytetracycline hydrochloride aerosol wound spray (Neo-caf, Intervet, Apilia, Italy) was applied on the incision. Dressing application was continued for one month for each rabbit.

Five mg/kg penrofloxacin (Baytril-K %5, Bayer, Leverkusen, Germany) was subcutaneously (SC) given pre-operatively and once daily post-operatively for five days. Each rabbit received 0.3-mg/kg butorphanol (Butomidor, Richter Pharma Ag, Austria) SC before the surgery and three consecutive days after the surgery for analgesic purposes^22^.

After the fourth post-operative week, the Steinmann pin osteosynthesis material was removed from all rabbit groups.

### Radiological examinations

Radiographs of the right hind leg of each patient were taken in the ML and anteroposterior (AP) positions after the operation and at the postoperative first, second, third, fourth, eighth, and 12th weeks. Radiographic images were evaluated and scored by three different specialists in terms of fracture healing, callus formation, and new bone formation. For the quantitative evaluation of radiographic images, guidance diagrams used by the researchers were considered.

After analysing the radiographic images, the amount of bone formation in the fracture side was scored in the longitudinal and transversal sections^23^^,^ 24, as shown in Table 1.

**Table 1:** Score values of radiographic images and lameness score values

Score	Radiographic image	Score	Variable
0	There is no callus formation	0	No signs of lameness were observed while walking
1	Bone formation fills < 25% of the fracture site	1	Mild lameness (there is vague lameness while walking)
2	Bone formation fills 25–50% of the fracture site	2	Moderate lameness (there is noticible lameness while walking)
3	Bone formation fills 50–75% of the fracture site	3	Severe degree of lameness (lameness prevents to put weight on)
4	Bone formation fills > 75% of the fracture site		
5	Bone formation fills the entire fracture area with clear boundaries		

Sources: Ayanoğlu et al.^24^ and Bergmann et al.^25^.

### Postoperative clinical examination and examination for lameness

In this study, right extremity lameness of the rabbits was observed after the removal of the pin and dressing material at the fifth, sixth, seventh, eighth, tenth, and 12th weeks. Findings of pain score, fever, lameness, and crepitation in the fracture side of the patients were documented. Indistinct lameness was defined as mild lameness. Lameness characterized by constant limping, but with the ability to bear weight, it was classified as moderate lameness, and cases in which the extremity could not be used at all or was held in a flexed position without any weight bearing were defined as severe lameness. Rabbits in which no signs of lameness were observed were evaluated and scored as completely healthy25. The score evaluation of the extremity functions and compression status of the rabbits was performed by three experts. The lameness of the rabbits was evaluated and classified as indicated in Table 1.

### Euthanasia of animals and collection of samples

Twelve weeks after the operation, a total of 40 rabbits in groups I, II, III, IV, and V were euthanized by administering 100 mg/kg of NA-pentobarbital (Penbital 400 mg, Bioveta, Ankara, Turkey). The right tibia bones of the rabbits were surgically removed by dissection and fixed in formol for histopathological examination.

### Histopathological examination

Hematoxylin and eosin, Masson trichrome, and Alcian blue-hematoxylin & orange G-eosin staining methods were used to examine fracture lines histopathologically. The samples were analyzed by comparing newly formed bone tissue, carilage tissue, fibrous tissue, and the extent of permanent damage. The bone samples were scored in four categories, and each category was evaluated on a scale of 0 to 10 (Table 2).

**Table 2:** Lesions examined at the fracture lines and their score equivalents during histopathological scoring.

Score	Newly formed bone tissue	Newly formed cartilage tissue	Newly formed fibrous tissue	Permanent damage size
0	No new bone formation was observed at the fracture line.	No cartilage tissue formation was observed at the fracture line.	Fibrous tissue was detected in ≤ %100 of the fracture line.	No new bone formation was observed at the fracture line (100٪ permanent damage area).
1	≤ %10 of the fracture line was fused with bone tissue.	≤ %10 of the fracture line was fused with cartilage tissue.	Fibrous tissue was observed in ≤ %90 of the fracture line.	≤ ٪10 of the fracture line was fused with new bone tissue.
2	≤ %20 of the fracture line was fused with bone tissue.	≤ ٪20 of the fracture line was fused with cartilage tissue.	Fibrous tissue was observed in ≤ %80 of the fracture line.	≤ %20 of the fracture line was fused with new bone tissue.
3	≤ %30 of the fracture line was fused with bone tissue.	≤ %30 of the fracture line was fused with cartilage tissue.	Fibrous tissue was observed in ≤ %70 of the fracture line.	≤ %30 of the fracture line was fused with new bone tissue.
4	≤ %40 of the fracture line was fused with bone tissue.	≤ ٪40 of the fracture line was fused with cartilage tissue.	Fibrous tissue was observed in ≤ ٪60 of the fracture line.	≤ %40 of the fracture line was fused with new bone tissue.
5	≤ %50 of the fracture line was fused with bone tissue.	≤ ٪50 of the fracture line was fused with cartilage tissue.	Fibrous tissue was observed in ≤ %50 of the fracture line.	≤ ٪50 of the fracture line was fused with new bone tissue.
6	≤ %60 of the fracture line was fused with bone tissue.	≤ ٪60 of the fracture line was fused with cartilage tissue.	Fibrous tissue was observed in ≤ %40 of the fracture line.	≤ ٪60 of the fracture line was fused with new bone tissue.
7	≤ %70 of the fracture line was fused with bone tissue.	≤ ٪70 of the fracture line was fused with cartilage tissue.	Fibrous tissue was observed in ≤ %30 of the fracture line.	≤ ٪70 of the fracture line was fused with new bone tissue.
8	≤ %80 of the fracture line was fused with bone tissue.	≤ ٪80 of the fracture line was fused with cartilage tissue.	Fibrous tissue was observed in ≤ %20 of the fracture line.	≤ ٪80 of the fracture line was fused with new bone tissue.
9	≤ %90 of the fracture line was fused with bone tissue.	≤ ٪90 of the fracture line was fused with cartilage tissue.	Fibrous tissue was observed in ≤ %10 of the fracture line.	≤ ٪90 of the fracture line was fused with new bone tissue.
10	%100 of the fracture line was fused with bone tissue (complete healing).	٪100 of the fracture line was fused with cartilage tissue.	No fibrous tissue was found on the fracture line (complete healing).	٪100 of the fracture line was fused with new bone tissue (complete healing).

Source: Elaborated by the authors.

- Newly formed bone tissue: score of 0 represents absence of any bone formation, and 10 indicates 90–100% of new bone formation;
- Newly formed cartilage tissue: score of 0 represents absence of any cartilage formation, and 10 indicates 90–100% new cartilage formation;
- Newly formed fibrous tissue: inverted scoring. Score of 10 represents no fibrous tissue, and 0 90–100% coverage;
- Extent of permanent damage: score of 10 indicates 90–100% fused with new bone, and 0 indicates absence of new bone fusing.

### Statistical evaluation

Before the study, a power analysis was performed using G*Power software to determine the appropriate sample size, aiming for the statistical power of 90%, effect size of 0.3, and three repeated measurements per participant across five groups.

Statistical analyses were carried out using the IBM Statistical Package for the Social Sciences Statistics 22.0 (IBM Corp., Armonk, New York, United States of America) program. The normal distribution of the data was evaluated with the Shapiro-Wilk test and Q-Q graphs. One-way analysis of variance (ANOVA) (alternative: Kruskal-Wallis test) was used for comparisons among groups. Repeated measures ANOVA (alternative: Friedman test, Student-Newman-Keuls) test was used for intertemporal comparisons. The post-hoc test Dunn-Bonferroni, and Nemenyi test were used. The relationship among categorical variables was examined with the exact method of the χ^2^ test. Lameness and X-ray scores were independently evaluated three times by the researcher and two board-certified veterinary surgeons (HE, MSC) from Erciyes University Department of Surgery.

To assess the reliability and consistency of these assessments, intraclass correlation coefficients were calculated using a two-way random effects model for absolute agreement, and values between 0.93 and 0.97 were obtained, indicating inter-rater reliability. The arithmetic mean of the obtained data was taken and used in statistical analysis. The intraclass correlation coefficients of the relationships among the researchers and the X-ray scores of the researcher and two other experts were found to be between 0.91–096. *p* < 0.05 was considered statistically significant. The data that follows a normal distribution and undergoes parametric testing is expressed as mean ± standard deviation, while data that does not follow a normal distribution is expressed in the results section as median ± minimum–maximum.

## Results

### Clinical findings

There was no abnormal finding in the general health status of the rabbits in the study. It was observed that the wound healing occurred between the eighth and the tenth day in rabbits in all groups, and the wound line was completely closed. In the study, clinical examination of the extremity was performed after removal of the pin and bandage materials on the 30th day after the operative intervention. After clinical examinations, local pain and bony crepitus findings were detected in a total of seven rabbits, four in group I, one in group II, one in group III, and one in group V. No signs of crepitation or pain at the fracture region were observed in four rabbits in the control group and 29 rabbits in the experimental groups.

### Radiological findings

**Figure 1:**
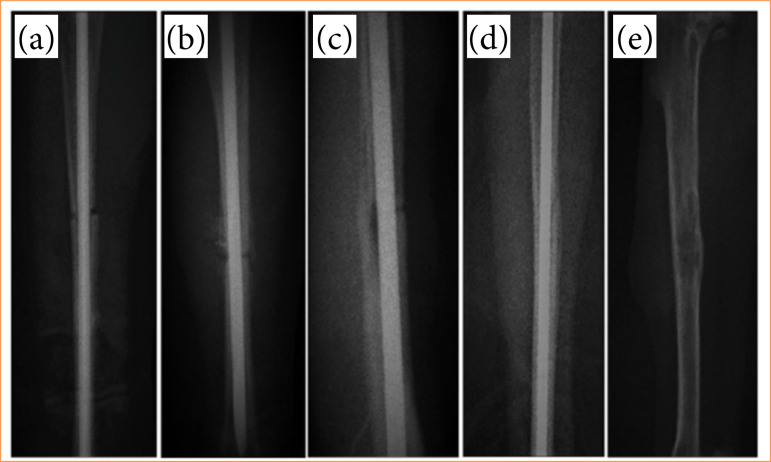
Radiographic images showing the healing process of the tibia fracture side. **(a)** Second week callus formation, x-ray score 1 (group III, T2); **(b)** third week callus formation, X-ray score 2 (group I, T5); **(c)** third week callus formation, x-ray score 3 (group V, T6); **(d)** fourth week callus formation, x-ray score 4 (Group II, T2); **(e)** eigthth week callus formation, x-ray score 5 (group IV, T3).

Source: Elaborated by the authors.

**Figure 2:**
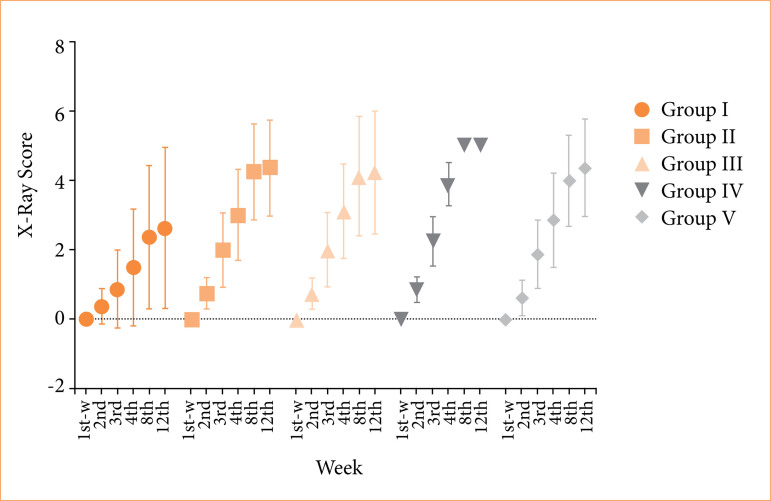
Comparison of x-ray scores among groups and according to time. The group with the highest scores was determined as group IV [fourth week score: 4 (3.25–4.0); eighth week score: 5 (5.0–5.0)]. In contrast, the group with the lowest score was the control group [fourth week score: 1.0 (0–3.0); eighth week score: 2.5 (0.25–4.0)]. These differences were statistically significant at weeks 4 and 8 (*p* = 0.031 and *p* = 0.008, respectively).

In this study, X-ray images obtained at postoperative weeks 1, 2, 3, 4, 8, and 12 [Figs. 1a (group III), 1b (group I), 1c (group V), 1d (group II), and 1e (group IV)] were evaluated and scored. The X-ray scores obtained were compared both by weeks over time and between groups (Fig. 2).

Group I: control group; Group II: laser group; Group III: bone marrow applied group; Group IV: β-tricalcium phosphate applied group; Group V: platelet-rich fibrin applied group. Source: Elaborated by the authors.

At the end of the fourth week, all experimental groups had higher X-ray scores compared to the control group. The highest score this week belonged to the group that received β-TCP [laser group: 3 (3.0–4.0); bone marrow treated group: 3.5 (3.0–4.0); PRF treated group: 3 (2.25–4.0)]. The data obtained at the eighth week showed a similar trend [laser group: 5.0 (4.0–5.0); bone marrow treated group: 5 (4.0–5.0); PRF treated group: 4 (4.0–5.0)].

These findings showed that β-TCP application was the most effective method to accelerate bone healing, and all of the experimental groups provided better radiologic healing compared to the control group.

### Findings of lameness

**Figure 3:**
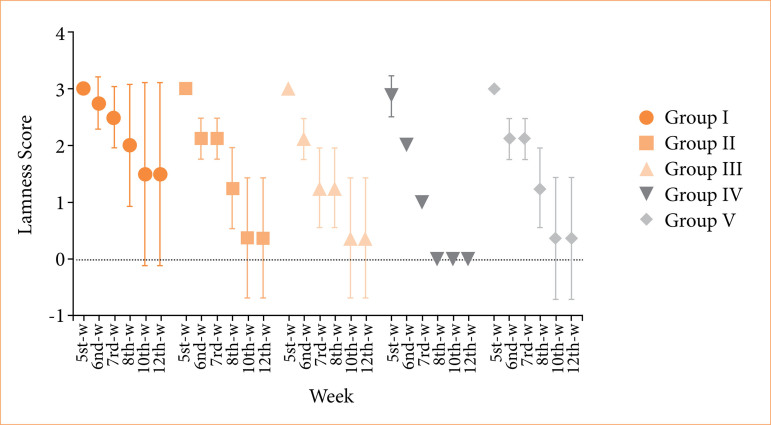
Comparison of lameness scores among groups and according to time. The highest limping scores were recorded in the control group [week 6: 3 (2.25–3.0); week 7: 2.5 (2.0–3.0); week 8: 2 (1.0–3.0)], while the β-tricalcium phosphate (β-TCP) group exhibited the lowest scores [week 6: 2 (2.0–2.0); week 7: 1 (1.0–1.0); week 8: 0 (0–0)]. These intergroup differences were statistically significant at all evaluated time points (week 6: *p* = 0.003; week 7 and 8: *p* < 0.001).

Assessment of postoperative limping was performed as described in the methods section. Four rabbits in group I and one rabbit in groups II, III, and V demonstrated persistent severe limping throughout the study, with complete non-use of the right hind limb. In contrast, all animals in group IV were able to bear weight on the affected limb by the end of the experimental period. Weekly distribution of lameness scores among and within the groups is given in Fig. 3. Lameness analysis started at week 5, since the extremities of rabbits remained in the cast for the first four weeks.

Group I: control group; Group II: laser group; Group III: bone marrow applied group; Group IV: β-tricalcium phosphate applied group; Group V: platelet-rich fibrin applied group. Source: Elaborated by the authors.

The laser, bone marrow, and PRF groups demonstrated intermediate scores, which were lower than those of the control group but higher than those observed in the β-TCP group [week 8 score: 1 (1.0–1.0)].

These findings suggested that β-TCP application significantly reduces the symptoms of claudication in the postoperative period, significantly supports functional recovery, and provides a more effective recovery compared to other treatment methods.

### Histopathological findings

**Figure 4:**
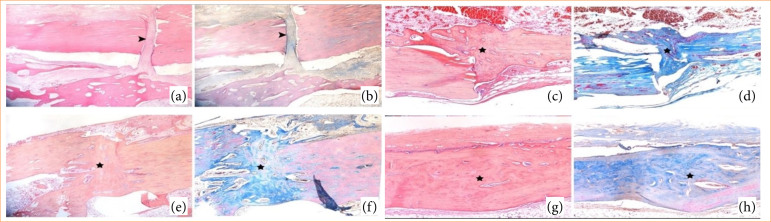
Fracture lines that healed to varying degrees in the study groups. (**a** and **b**) Soft callus formation characterized by fibrotic growths outward from the fracture line in a sample in group V (arrowhead). (**c** and **d**) Hard callus formation characterized by new bone and cartilage tissue formations at the fracture line (star) in an example in group II. In a sample in group III, (**e** and **f**) new bone tissue formation at the fracture line was more pronounced, and a hard callus formation can be seen (star). In a sample in group IV, (**g** and **h**) complete healing with new bone tissue formation at the fracture line is seen (star). (**a**, **c**, **g**, and **e**) Alcian blue-hematoxylin & orange G-eosin staining; (**b**, **d**, **f,** and **h**) Masson trichrome staining.

Source: Elaborated by the authors.

No sign of fracture healing was detected histopathologically in seven rabbits in all groups (group I: four rabbits, one in groups II, III, and V). Only one animal was documented to have a complete recovery (group IV, T4). While new bone tissue formations were found at the fracture line in 27 rabbits (group I: four rabbits, group II: six rabbits, group III: six rabbits, group IV: seven rabbits, group V: four rabbits), cartilage tissue was found in the formed callus in 25 of them (group I: four rabbits, group II: four rabbits, group III: six rabbits, group IV: five rabbits, group V: six rabbits). Fibrotic growths at the fracture line were found in 17 animals (group I: six rabbits, group II: three rabbits, group III: three rabbits, group IV: two rabbits, group V: three rabbits). Pictures of different stages of fracture line healing are given in Fig. 4.

**Figure 5:**
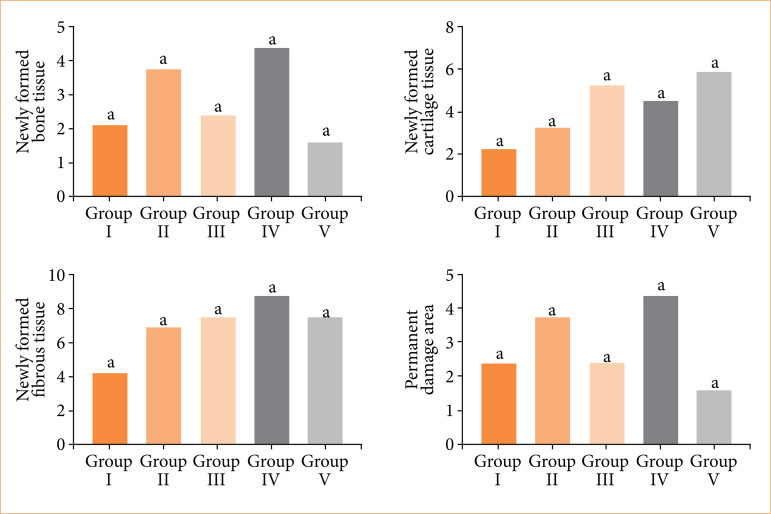
Histopathological score averages of the study groups (newly formed fibrous tissue calculated with instead of scoring).

Histopathological analysis revealed variation in fracture healing among the experimental groups. Group IV (β-TCP applied group) demonstrated the highest mean score (22.13 ± 4.82), primarily due to consistent hard callus formation and one case of complete bone regeneration. Group III (bone marrow applied group) and group II (laser group) followed with mean scores of 17.63 ± 8.12 and 17.75 ± 9.66, respectively, both characterized by predominant hard callus development. Group V (PRF applied group) exhibited a lower mean score of 16.63 ± 8.88, reflecting fewer instances of hard callus. The control group (group I) had the lowest healing outcomes (mean score: 11.13 ± 12.43), with no healing in four cases. The comparative histological scores across groups are illustrated in Fig. 5.

Group I: control group; Group II: laser group; Group III: bone marrow applied group; Group IV: β-tricalcium phosphate applied group; Group V: platelet-rich fibrin applied group. Source: Elaborated by the authors.

In our study, the most pronounced new bone formation and the fastest healing process were observed in group IV. This group demonstrated the highest histopathological score (22.13 ± 4.82), the greatest extent of new bone tissue formation (4.37 ± 3.34), and complete fracture healing. These findings indicated that the combined β-TCP treatment is the most effective method for promoting fracture repair. Additionally, group IV exhibited the highest score for new fibrous tissue formation (8.88 ± 1.46), indicating the lowest level of fibrous tissue presence, which is associated with more favorable healing. In contrast, group I had the lowest performance across all evaluated parameters, including the lowest histopathological score (11.13 ± 12.43), minimal new bone formation (1.62 ± 1.77), the lowest fibrous tissue score (4.38 ± 4.96), and the highest number of unhealed fractures.

**Figure 6:**
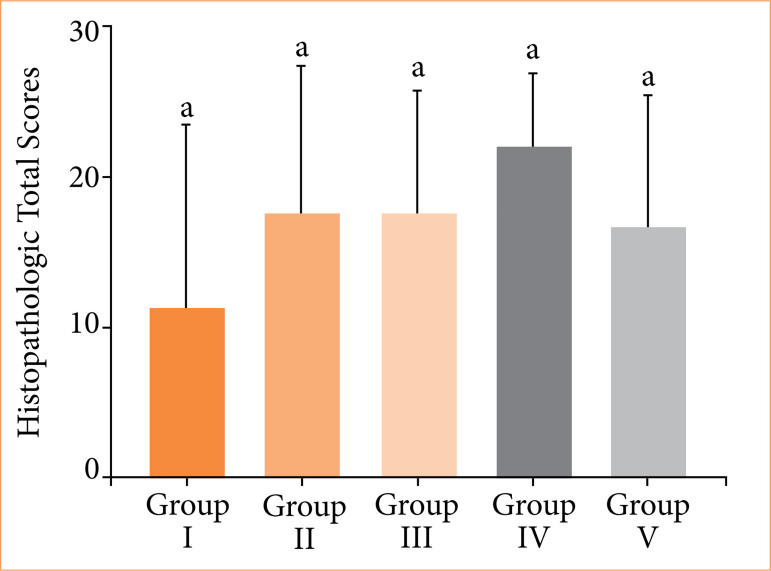
Statistical analysis of the histopathological score differences of the groups. The scores were 11.13 ± 12.43 in group I, 17.75 ± 9.66 in group II, 22.13 ± 4.82 in group IV, 16.63 ± 8.88 in group V, and 17.63 ± 8.12 in group III. The differences between the groups were not statistically significant (*p* = 0.227).

Groups II, III, and V showed comparable histological scores and similar levels of fibrous tissue and callus formation. However, new bone formation was more prominent in group II (3.75 ± 3.24) compared to groups III (2.38 ± 2.20) and V (1.62 ± 1.77), with group V showing the least bone regeneration among the experimental groups. Statistical analysis and graph of histopathological scores are given in Fig. 6.

Group I: control group; Group II: laser group; Group III: bone marrow applied group; Group IV: β-tricalcium phosphate applied group; Group V: platelet-rich fibrin applied group. Source: Elaborated by the authors.

The findings of this study demonstrated that the combined β-TCP treatment (group IV) significantly enhanced fracture healing by promoting greater new bone formation and achieving higher histopathological scores, while the untreated control group (group I) exhibited the poorest regenerative outcomes across all evaluated parameters.

## Discussion

In this study, the combination of bone marrow aspirate and β-TCP was shown to significantly increase bone regeneration. Group IV showed the highest new bone formation, the lowest fibrotic tissue development, and the best histopathologic and radiographic results. In addition, functional recovery was the fastest in this group, and complete healing was achieved at week 8.

Our findings agreed with previous research. Szabo et al.^26^ reported that, although β-TCP resorbs slowly, it effectively stimulates new bone formation. Similarly, Wang et al.^27^ and Jarcho^28^ suggested that β-TCP is a biodegradable material that creates a favorable microenvironment for osteogenesis by releasing high-local concentrations of calcium and phosphate upon degradation. Becker et al.^29^ also demonstrated in an ovine tibial defect model that β-TCP ceramics combined with bone marrow yielded the most robust bone regeneration, and they concluded that such combinations exhibit osteopromotive effects when implanted. Consistent with these findings, our results indicated that the synergistic use of bone marrow aspirate with β-TCP provides a highly effective strategy for enhancing bone healing.

Krzymanski et al.^17^ investigated the regenerative potential of various graft materials in rabbit mandibular bone defects. Their findings indicated that both fresh autologous bone grafts and bone marrow aspirates yielded comparable and effective outcomes in defect closure. Notably, significant bone regeneration was also achieved using bone marrow aspirate alone, highlighting its osteogenic potential. Similarly, Connolly et al.^30^ demonstrated that the injection of bone marrow aspirate into non-union tibial defects in rabbits significantly enhanced osteogenesis, with notable radiographic signs of healing observed in five weeks.

In alignment with these findings, our study showed that the group treated solely with fresh bone marrow aspirate exhibited higher histopathological and radiological scores compared to the control group. This suggests that graft material containing viable cells has a considerable capacity to fill fracture gaps and act as a functional bone matrix. However, these results contrast with the opinions of some researchers who argue that bone marrow aspirate alone may not serve as a reliable or sufficiently potent primary source for bone regeneration^31^^,^ 32. Variability in the cellular content of the aspirated marrow, as well as differences in the anatomical location and type of bone used in experimental models, may contribute to divergent outcomes in the literature^33^. Furthermore, it is important to acknowledge that osteogenic cells are not the sole determinant of bone healing, and multiple biological and biomechanical factors likely influence the success of different treatment modalities^34^.

Low-energy laser therapy has been widely reported to exert beneficial effects on bone tissue by enhancing mitochondrial activity, nucleic acid synthesis, cell viability, and osteoblastic function^29^^,^ 35 ^,^ 36. Consistent with these findings, the current study observed improved bone healing outcomes, evidenced by higher histopathological, radiological, and clinical (lameness) scores in the laser-treated group compared to controls. These results support the hypothesis^37^ that laser therapy facilitates osteogenic differentiation of mesenchymal stem cells and accelerates fracture repair by promoting early-stage bone regeneration.

Several studies^39^^–^ 41, including the one by Nagata et al.^38^, reported that low-energy laser therapy does not significantly enhance bone regeneration. In their rat model with a 5-mm cranial defect, laser application (660 nm, 4.9 J/cm²) showed no difference from the control group. Similarly, other investigations have documented a lack of beneficial effects on bone healing and suggested that low-energy lasers may not exert a biomodulatory influence on bone tissue^39^^,^ 42.

In contrast to studies reporting no effect of low-energy laser on bone healing, the present study demonstrated that laser application (830 nm, 10 J/cm^2^) over 30 days postoperatively significantly improved fracture healing in the tibia. Histopathological, radiological, and lameness assessments consistently indicated superior outcomes compared to the control group, with the laser group ranking second among experimental treatments. These discrepancies are likely attributable to differences in laser parameters, including wavelength, energy density, application duration, and delivery mode, highlighting the importance of optimizing laser protocols for effective bone regeneration.

This study demonstrated that PRF application enhanced bone healing compared to the control group, as evidenced by increased bone volume on radiographs, higher histopathological scores, and improved lameness outcomes. However, the extent of new bone formation in the PRF group remained lower than in other experimental groups, indicating a relatively limited osteogenic potential. Faot et al.^21^ reported that PRF did not significantly enhance bone healing in rabbit tibial defects during a short-term period (14–28 days). In contrast, Kim et al.^43^ demonstrated increased bone volume and new bone formation in rabbit calvarial defects treated with PRF at both six and 12 weeks postoperatively. In contrast to the short-term findings by Faot et al.^21^, our 12-week evaluation shows that PRF membranes enhance bone healing, especially in the later stages of repair. These results are evidenced by improvements in radiologic, histopathologic, and functional outcomes consistent with the findings of Kim et al.^43^.

In our study, the PRF group showed moderate improvement in bone healing with histopathological scores lower than the laser and bone marrow groups but higher than the control group. While the β-TCP combination group demonstrated the highest scores, these results were supported by radiographic and functional assessments. Abdullah et al.^44^ demonstrated that the combination of PRF and β-TCP had a synergistic effect on bone regeneration, significantly enhancing new bone formation and improving the structural integrity of the defect site. They documented that the bone healing results were higher in the use of the PRF graft material than in the control group, but lower than the combined use of β-TCP. In contrast, studies by Pradeep et al.^45^ and Ajwani et al.^46^, which applied the PRF membranes to intraosseous defects, reported no statistically significant improvement in bone regeneration. Contrasting findings indicated that the effectiveness of PRF membrane may vary depending on the application method and experimental model. These results suggest that PRF membrane may promote bone regeneration, but further combined analyses are needed to evaluate its therapeutic potential.

There are some limitations regarding the generalizability and direct applicability of the current study results to humans. The study was performed on the rabbit tibia. Although this model shows biological processes similar to human bone healing, anatomical and physiological differences may limit the direct transfer of results to humans. The limited number of subjects used and the evaluation of healing for only eight–12 weeks does not provide sufficient information on long-term effects and complications. Conflicting results in the literature, especially for low-energy laser applications, are due to different wavelengths, dosages, and application times. This makes it difficult to make definitive judgments about laser efficacy.

The study found that combining β-TCP with bone marrow aspirate significantly enhances bone regeneration via a synergistic effect, indicating potential for advanced surgical reconstruction and critical bone defect treatments. Bone marrow aspirates’ effectiveness depends on their cellular content, supporting the need for phase I/II clinical trials to assess this combination in humans. Additionally, further systematic research is required to clarify laser therapy’s efficacy due to variable parameters. Given PRF’s limited effect alone but synergistic potential with osteoconductive materials, testing various combinations *in vitro* and *in vivo* is recommended. Overall, the study offers valuable preclinical insights and supports the continued exploration of multimodal treatment strategies in translational bone regeneration research.

## Conclusion

The combination of aspirated bone marrow and β-TCP granules showed the highest bone regeneration in complete fractures, while PRF membrane demonstrated the least osteogenic effect. This study contributes to the evaluation of current treatment methods and highlights the need for larger controlled studies to guide clinical application and standardization.

## Data Availability

The data will be available upon request.
